# Magnitude and determinants for place of postnatal care utilization among mothers who delivered at home in Ethiopia: a multinomial analysis from the 2016 Ethiopian demographic health survey

**DOI:** 10.1186/s12978-019-0818-2

**Published:** 2019-11-08

**Authors:** Brhane Gebrekidan Ayele, Mulugeta Abrha Woldu, Haftom Weldearegay Gebrehiwot, Equbay Gebru Gebre-egziabher, Hailay Gebretnsae, Tsegay Hadgu, Alemnesh Araya Abrha, Araya Abrha Medhanyie

**Affiliations:** 1 Tigray Health Research Institute, Mekelle, Ethiopia; 2 Department of Midwifery, College of Health Sciences, Mekelle University, Mekelle, Ethiopia; 3 College of Health Sciences, School of Public Health, Mekelle University, Mekelle, Ethiopia

**Keywords:** Postnatal care, Home delivery, Maternal service utilization, Place for PNC, Ethiopia

## Abstract

**Introduction:**

Above half of mothers in Ethiopia give birth at home. Home based care within the first week after birth as a complementary strategy to facility-based postnatal care service is critical to increase the survival of both mothers and newborns. However, evidence on utilization of postnatal care and location of service among mothers who delivered at home in Ethiopia is insufficiently documented. Therefore, this study assessed the magnitude and determinants for place of postnatal care service utilization among mothers who delivered at home in Ethiopia.

**Methods:**

We used the 2016 Ethiopian Demographic and Health Survey, and extracted data from 4491 mothers who delivered at home during 5 years preceding the survey. A multinomial logistic regression model was applied to examine the determinants of both facility and home -based postnatal care service utilization. Likelihood ratio test was used to see the model fitness and *p*-value of < 0.05 was used to determine statistical significance at 95% confidence interval.

**Results:**

From the total 4491 mothers who delivered at home, only 130(2.9%) and 236(5.3%) of them utilized postnatal service at home and at a health facility respectively. Being from an urban region (AOR = 0.378, 95%CI: 0.193–0.740), ever using the calendar method to delay pregnancy (*AOR* = 0.528, 95%CI: 0.337–0.826), receiving four and above antenatal care visits (*AOR* = 0.245, 95%CI: 0.145–0.413) and having a bank account (*AOR* = 0.479, 95%CI: 0.243–0.943) were the factors associated with utilizing home- based postnatal care. Similarly being a follower of the orthodox religion (*AOR* = 1.698, 95%CI: 1.137–2.536), being in the rich wealth index (*AOR* = 0.608, 95%CI: 0.424–0.873), ever using the calendar method to delay pregnancy (*AOR* = 0.694, 95%CI: 0.499–0.966), wantedness of the pregnancy (*AOR* = 0.264, 95%CI: 0.352–0.953), receiving four and above antenatal care visits (*AOR* = 0.264, 95%CI: 0.184–0.380) and listening to radio at least once a week (*AOR* = 0.652, 95%CI: 0.432–0.984) were the determinants of facility-based postnatal care utilization.

**Conclusion:**

The coverage of postnatal care service utilization among mothers who delivered at home was very low. Living in urban region, following the Orthodox religion, having higher wealth index, having a bank account, ever using calendar method to delay pregnancy, wantedness of the pregnancy, receiving four and above antenatal care visit and listening to radio at least weakly were associated with postnatal care service utilization. Therefore, targeted measures to improve socio-economic status, strengthen the continuum of care, and increase health literacy communication are critically important to increase postnatal care service utilization among women who deliver at home in Ethiopia.

## Plain English summary

Postnatal care is the care given to the mother and her newborn baby immediately after the birth and for the first 6 weeks postpartum irrespective of place of delivery. In Ethiopia evidence on utilization of postnatal care for mothers who delivered at home is insufficiently documented. Using national health survey data, this study sought to illuminate the magnitude, location and factors associated with postnatal care utilization among mothers who deliver at home in Ethiopia.

Data on mothers who delivered at home within the 5 years preceding the survey were taken from the nationally representative dataset. Accordingly 4491 mothers were included in the analysis.

The coverage of postnatal care service utilization among home delivered mothers was very low. In this study only 366(8.2%) of mothers who delivered at home received postnatal care. Living in an urban region, following the Orthodox religion, being in a high wealth index, having a bank account, ever used calendar to delay pregnancy, wantedness of the pregnancy, receiving four or more antenatal care visits and listening to radio at least weakly were associated with postnatal care utilization.

Targeted measures to improve socio-economic status, and strengthen awareness of postnatal care service and health literacy communications about postnatal care are critically important to increase postnatal care service utilization among women delivering at home.

## Introduction

The early postnatal period, particularly the first hours of life, extending into the first 2–3 days of life is a period of high risk for both mothers and newborns [1–3]. The risk is more severe in communities where the overwhelming majority of births occur at home [4, 5]. In such communities unless safe household practices are followed and care is provided by health workers, undoubtedly catastrophic adverse outcomes will persist [1, 6]. Based on evidence, the World Health Organization (WHO) recommends home-based postnatal care (PNC) care for mothers and newborns in the first week of life as a complementary strategy to facility-based PNC in order to improve mothers and newborns survival [1, 7, 8].

Studies had shown that home-based newborn care interventions can prevent 30–61% of newborn deaths in high mortality settings and can lower the odds of maternal postpartum distress [9].

A home-based care strategy to promote an integrated package of preventive and curative newborn care is effective in reducing neonatal mortality in communities, even within a weak health system, especially if it is given by trained health professionals such as midwives [6, 9]. However, providing this service and ensuring optimal practices is not straightforward for many reasons such as staff shortages, and inconvenient transportation, which results in low to moderate home visitation coverage for PNC even with intensive support [1, 7]. Though they do not replace antenatal care (ANC), home visits by the community health workers during pregnancy were higher than the visits after birth [1, 10–12].

For institutional births, opportunities already exist to provide PNC to mothers and newborns before discharge [1]. However, even at health facility level, in Sub-Saharan Africa women are often discharged before 24 hrs, which limits them from receiving the WHO’s recommended services. Additionally visits at 72 hrs and seventh day are rare [13]. For home births, some mothers and babies access early postnatal care through a visit to a health facility. However, many cannot or do not receive facility-based PNC following home deliveries, leaving home visitation as a potentially attractive way to make such care available [1].

In Ethiopia, the maternal mortality ratio is still high; 412 maternal deaths out of 100,000 live births. Maternal mortality ratio has been reduced over the past two decades though still it remains high, with most maternal deaths happening during the postpartum period [14, 15]. The country has a significant proportion (above half, 52%) of home deliveries, and has been implementing PNC through home visitation as one of the interventions to reduce maternal and child morbidity and mortality [1, 16]. In Ethiopia, the Health Management Information System (HMIS) has indicators used to monitor the implementation of PNC for mothers and babies, which includes both health facility and home visits [7]. However, PNC coverage is very low with 42–48% of mothers delivering in a health facility receiving PNC, and only 1–2% of women delivering at home receiving PNC in the first 2 days of postpartum period [1, 14].

The main reasons listed for low PNC utilization in studies of different countries range from low awareness about the need to fear of health facilities [17, 18]. Furthermore mothers who deliver at home may experience discrimination in receiving PNC due to some locally practiced measures like financial penalties for home births [18, 19]. Moreover; the perspective of women, their families and community on the quality of service could influence their decision whether or not to seek care [20, 21].

Maternal, newborn and child health issues are national priorities of many countries. However, specific policies for postpartum care are weak, and there is a very little evidence of effective PNC implementation [7, 13]. The ideal way to provide maternal and child health services such as PNC is through health services delivered by skilled personnel in health facilities. However, in developing countries like Ethiopia, a more realistic model requires working hard to strengthen the health system and improve access to facility-based care, while also enabling access to PNC at the community level through home visit [13, 22].

Comprehensive studies on utilization of PNC through facility-based and home-based care are rare. However, having such inclusive evidence is critically important to develop appropriate strategies to help improve the low service utilization and reduce the maternal and newborn morbidity and mortality, especially if the evidence is derived from country specific data like the Demographic Health Surveys. Therefore, this study aimed to assess the utilization of PNC and its determinants among mothers who delivered at home in Ethiopia.

## Methods

### Data source and study design

This was a cross-sectional secondary analysis of data collected in the 2016 Ethiopian Demographic Health Survey (EDHS). It is the fourth EDHS conducted by the Central Statistical Agency as part of the International Demographic and Health Survey program. The data for mothers who delivered at home were extracted from the pregnancy and PNC dataset of the EDHS 2016 and included. We only included the most recent child of the women so as to avoid mix-ups in the recall and reporting of mothers experiences, especially for mothers who had more than one birth in the previous 5 year period. Additionally, mothers who did not remember the PNC they received for either the mother herself or her newborn were excluded from analysis. Therefore, a total of 4491 mothers (between 15 and 49 years) were included in this study.

### Outcome variable

To develop the outcome variable from the dataset the question “where did you give birth?” was used as a starting point for this study. According to this question the dataset is categorized in to two subgroups i.e. “1=health facility delivery” and “2 = home delivery”. Accordingly, from the total (*N* = 7188) mothers, 37.5% (*n* = 2697) delivered at a health facility and 62.5% (*n* = 4491) of them gave birth at home. The latter subgroup was used as our study participants in this study. Using the responses to the questions “Did anyone check on your health after you gave birth?” and “In the two months after delivery, did anyone check the health of your child?” we determined how many mothers and newborns received postnatal care services. According to the WHO, PNC service is defined as service given for both the mother and her newborn within 42 days of delivery [2]. However, the EDHS 2016 dataset contained data for care provided to newborns extending up-to 2 months. This does not contradict to the WHO’s definition rather it is better to give comprehensive understanding on the community based newborn care (CBNC) program which is being implemented as an intervention to decrease the high ratio of newborn deaths in the country [23].

For mothers who indicated that they had been “Checked by anyone”, we used the questions “Where did your first health check take place?” and “Where did your child’s first health check take place?” Responses categorized “at health facility” included those who were checked at governmental, private and NGO health facilities and “at home” was used to define those receiving PNC at home. Since PNC includes services for both the newborn and the mother, we consider checked at home and health facility for either of the two (mother or her newborn) or both for those who were checked by Doctor, Nurse, Midwife, Health Officer or Health Extension Worker using the data on the questions “who checked your health at that time?” and “who checked your child’s at that time?”. Respondents who were checked by traditional birth attendants and other family members were considered as not receiving PNC.

The outcome variable was “place of PNC utilization” which was categorized in to three levels: 1) At home when either the mother or newborn or both were checked at home by a health personnel after giving birth at home, 2) At health facility when either of mother or newborn or both were checked at health facility after giving birth at home and 3) Not utilized at all when neither the mother nor newborn were checked by a trained personnel either at home or at a health facility a giving birth at home.

### Independent variables

The independent variables included socio-demographic factors (age, marital status, educational status, place of residence, region type, religion, number of family members and sex of family head), socio-economic factors (wealth status, respondent’s and husband’s occupation, having a bank account and autonomy for health service utilization) and maternal health service, and health literacy communication related factors (wantedness of the last child, ever using anything to delay pregnancy, ANC utilization and frequency, awareness on fistula, owning a mobile phone and frequency of listening to radio).

### Data analysis

Frequencies and proportions were used to describe categorical variables using cross tabulation. Multi-collinearity was checked using Variance Inflation Factor (VIF) test and variables with value of > 5 were excluded from the model. Multinomial logistic regression model was applied to identify determinants of place of postnatal care service utilization. Likelihood ratio test was used to see the model fitness and the value was 0.000 which showed the model was well-fitted. The adjusted odds ratio (AOR) was reported with their 95% confidence interval, and a *p*-value of < 0.05 was considered to declare statistical significance. The overall correct predicted classification of the model in this analysis was 91.9%.

## Results

### Socio-demographic characteristics

Among young mothers (15–24 years of age) who delivered at home, only 4.5% (*n* = 48) utilized PNC at a health facility and more than 90% (*n* = 980, 92.9%) did not utilize PNC at all. Among urban and rural residents, 14% (*n* = 36) and 7.8% (*n* = 330) utilized PNC service at a health facility or home respectively. A slightly higher percentage of mothers with 1–4 family members utilized PNC either at home or at a health facility (8.4%) compared to mothers with > 8 number of family members (6.7%) **(**Table 1**).**

**Table 1:** Socio-demographic characteristics of study women by place of postnatal care service utilization in Ethiopia, analysis from EDHS 2016

Variable	Place of PNC Utilization			
	At Home n (%)	At Health Facility n (%)	Not utilized at all n (%)	Total N (%)
Age group				
15–24 years	27 (2.6)	48 (4.5)	980 (92.9)	1055 (100)
25–34 years	70 (3.2)	120 (5.5)	2001 (91.3)	2191 (100)
35–49 years	33 (2.7)	68 (5.5)	1144 (91.8)	1245 (100)
Marital status				
Married/lived w p.	123 (2.9)	216 (5.1)	3872 (91.6)	4211 (100)
Others	7 (2.5)	20 (7.1)	253 (90.4)	280 (100)
Educational status				
Not Educated	91 (2.7)	149 (4.4)	3152 (92.9)	3392 (100)
Primary and above	39 (3.5)	87 (7.9)	973 (88.5)	1099 (100)
Religion				
Orthodox Christian	40 (3.5)	103 (9.1)	991 (87.4)	1134 (100)
Muslim	61 (2.6)	86 (3.7)	2166 (93.6)	2313 (100)
Others	29 (2.8)	47 (4.5)	968 (92.7)	1044 (100)
Place of residence				
Urban	8 (3.1)	28 (10.9)	220 (85.9)	256 (100)
Rural	122 (2.9)	208 (4.9)	3905 (92.2)	4235 (100)
Region				
Agrarian	62 (2.8)	149 (6.7)	2019 (90.5)	2230 (100)
Pastoralists	52 (2.7)	65 (3.3)	1824 (94)	1941 (100)
Urban administration	16 (5.0)	22 (6.9)	282 (88.1)	230 (100)
No family members				
1–4	33 (2.7)	68 (5.7)	1101 (91.6)	1202 (100)
5–7	65 (2.9)	128 (5.8)	2023 (91.3)	2216 (100)
> = 8	32 (3.0)	40 (3.7)	1001 (93.3)	1073 (100)
Sex of household head				
Male	106 (3.0)	192 (5.4)	3266 (91.6)	3564 (100)
Female	24 (2.6)	44 (4.7)	859 (92.7)	927 (100)
Partner’s Educational level				
No education	56 (2.2)	109 (4.3)	2360 (93.5)	2525 (100)
Primary	53 (4.0)	86 (6.5)	1180 (89.5)	1319 (100)
Secondary and above	14 (3.8)	21 (5.7)	332 (90.5)	367 (100)

*p* partner, *Educ* Educational

### Socio-economic characteristics

A higher proportion of mothers in the highest wealth index utilized PNC either at home or health facility than participants in the lowest wealth index (14.4 and 5.5% respectively). Furthermore, mothers with a bank account were more likely to utilize PNC either at health facility or home compared to participants without bank account (20.8% (*n* = 38) and 7.6% (*n* = 328) respectively) **(**Table 2**).**

**Table 2:** Socio-economic characteristics of study women by place of postnatal care service utilization in Ethiopia, analysis from EDHS 2016

Variable	Place of PNC Utilization			
	At Home n (%)	At Health Facility n (%)	Not utilized at all n (%)	Total N (%)
Wealth Index				
Poorer	62 (2.1)	100 (3.4)	2746 (94.4)	2908 (100)
Middle	31 (4.4)	46 (6.6)	625 (89.0)	702 (100)
Richer	37 (4.2)	90 (10.2)	754 (85.6)	8891 (100)
Husband’s occupation				
Agricultural work	85 (3.1)	131 (4.9)	2483 (92.0)	2699 (100)
Others	38 (2.5)	85 (5.6)	1389 (91.9)	1512 (100)
Participant’s occupation				
No work	65 (2.4)	102 (3.8)	2534 (93.8)	2701 (100)
Agricultural work	35 (3.3)	73 (6.9)	943 (89.7)	1051 (100)
Others	30 (4.1)	61 (8.3)	648 (87.7)	739 (100)
Autonomy to utilize PNC				
Self	23 (3.5)	41 (6.2)	602 (90.4)	666 (100)
Self and partner	70 (2.7)	134 (5.2)	2380 (92.1)	2584 (100)
Partner alone	29 (3.0)	41 (4.3)	882 (92.6)	952 (100)
Others	8 (2.8)	20 (6.9)	261 (90.3)	289 (100)
Has bank account				
No	117 (2.7)	211 (4.9)	3981 (92.4)	4309 (100)
Yes	13 (7.1)	25 (13.7)	144 (79.1)	182 (100)

### Health service and health literacy communication related characteristics

About 12% (*n* = 105) of mothers who had received 4+ ANC visits utilized PNC at health facility, while only 2.4% (*n* = 55) of mothers who did not receive ANC utilized PNC at health facility. Furthermore 19% (*n* = 148) of mothers who were told about pregnancy danger signs during their ANC visit utilized PNC at home or a health facility, compared to only 10% (*n* = 139) of mothers who were not counseled on danger signs during ANC. A higher proportion of mothers who were aware of fistula utilized PNC at home or health facility compared to those who were not aware of fistula (12.1 and 6.7% respectively) **(**Table 3**).**

**Table 3:** Maternal health service and health literacy communication related characteristics of study women by place of postnatal care service utilization in Ethiopia, analysis from EDHS 2016

Variable	Place of PNC Utilization			
	At Home n (%)	At Health Facility n (%)	Not utilized at all n (%)	Total N (%)
Number of ever born children				
1–2 children	32 (2.6)	64 (5.3)	1112 (92.1)	1208 (100)
3–4 children	37 (3.0)	72 (5.8)	1143 (91.3)	1252 (100)
> = 5 children	61 (3.0)	100 (4.9)	1870 (92.1)	2031 (100)
Number of living children				
4 and bellow	77 (2.8)	158 (5.7)	2536 (91.5)	2771 (100)
5 and above	53 (3.1)	78 (4.5)	1589 (92.4)	1720 (100)
Ever used anything to delay pregnancy				
No	53 (1.9)	88 (3.1)	2683 (95.0)	2824 (100)
Used outside calendar	8 (6.2)	13 (10.1)	108 (83.7)	129 (100)
Used in calendar	69 (4.5)	135 (8.8)	1334 (86.7)	1538 (100)
Wanted pregnancy when become pregnant				
Then	96 (2.7)	159 (4.4)	3350 (92.9)	36.5 (100)
Later	28 (4.9)	53 (9.2)	495 (85.9)	576 (100)
No more	6 (1.9)	24 (7.7)	280 (90.3)	310 (100)
Number of ANC Visits				
No ANC visit	25 (1.1)	55 (2.4)	2229 (96.5)	2309 (100)
1–3 visits	58 (4.6)	76 (6.0)	1137 (89.5)	1137 (89.5)
> =4 visits	47 (5.2)	105 (11.5)	759 (83.3)	911 (100)
Told about pregnancy danger signs				
Have no ANC visit	24 (1.0)	55 (2.4)	2219 (96.6)	2298 (100)
Not told	50 (3.6)	89 (6.3)	1264 (90.1)	1403 (100)
Told	56 (7.1)	92 (11.6)	642 (81.3)	790 (100)
Told about birth preparedness plan				
No ANC visit (N/A)	24 (1.0)	55 (2.4)	2219 (96.6)	2298 (100)
No	41 (3.4)	66 (5.5)	1102 (91.1)	1209 (100)
Yes	65 (6.6)	115 (11.7)	804 (81.7)	984 (100)
Size of newborn at birth				
Larger than average	35 (2.8)	61 (4.9)	1144 (92.3)	1240 (100)
Average	59 (3.1)	105 (5.6)	1720 (91.3)	1184 (100)
Small than average	36 (2.6)	70 (5.1)	1261 (92.2)	1367 (100)
Ever heard of fistula				
No	77 (2.3)	143 (4.4)	3062 (93.3)	3282 (100)
Yes	53 (4.4)	93 (7.7)	1063 (87.9)	1209 (100)
Owns mobile telephone				
No	113 (2.9)	190 (4.8)	3644 (92.3)	3947 (100)
Yes	17 (3.1)	46 (8.5)	481 (88.4)	544 (100)
Frequency of listing to radio				
Not at all	94 (2.5)	165 (4.5)	3448 (93.0)	3707 (100)
Less than once a week	19 (4.6)	34 (8.2)	361 (87.2)	414 (100)
At least once a week	17 (4.6)	37 (10.0)	316 (85.4)	370 (100)

*N/A* Not Applicable

### Magnitude of PNC utilization

**Fig. 1:**
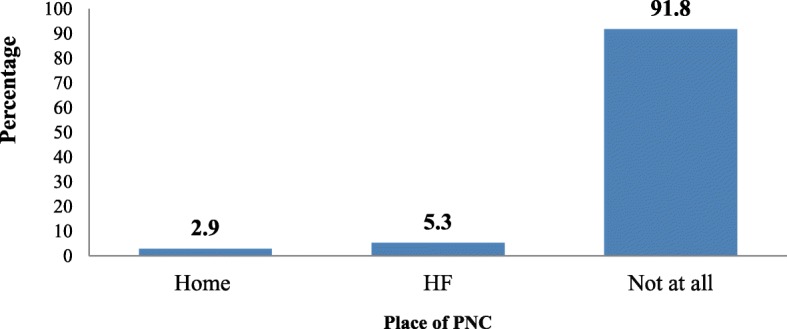
Place of postnatal care utilization among home delivered mothers in Ethiopia, analysis of EDHS 2016

From the total 4491 mothers who delivered at home included in the study, 130 (2.9%) utilized postnatal service at home, while 236 (5.3%) of them utilized PNC at a health facility (Fig. 1).

### Factors affecting postnatal care utilization

Type of region (agrarian, pastoralist or urban) where mothers live, ever using anything to delay pregnancy, receiving ANC and having a bank account were all significant factors affecting home-based postnatal care utilization. Participants from agrarian regions were 62.2% less likely to receive PNC at home than not utilizing at all when compared to participants from urban areas (AOR = 0.378, 95%CI 0.193–0.740). Participants who had not ever used anything to delay pregnancy were 47.2% less likely (AOR = 0.528, 95%CI 0.337–0.826) to receive PNC at home than not utilizing at all when compared to those used calendar as method of delay to pregnancy. Furthermore participants with > 4 ANC visits were 4 times (AOR = 4.1, 95%CI 2.42–6.88) more likely to receive PNC at home than not utilizing at all when compared to those who have no ANC visit. Participants without a bank account were approximately half as less likely (AOR = 0.479, 95%CI 0.243–0.943) to receive PNC at home when compared to participants with a bank account (Table 4).

**Table 4:** Coefficients of multinomial logistic regression on place of PNC utilization in Ethiopia, analysis from EDHS 2016

Predictor variables	Multinomial Logit Model							
	Utilizes PNC at home versus not utilized at all				Utilizes PNC at HF versus not utilized at all			
			95%CI				95%CI	
	*P*-value	AOR	Lower bound	Upper bound	*P*-Value	AOR	Lower bound	Upper bound
Age group								
15–24 years	0.571	1.241	0.588	2.618	0.306	0.745	0.423	1.310
25–34 years	0.366	1.250	0.770	2.030	0.458	0.868	0.596	1.262
35–49 years ^RC^								
Region								
Agrarian^a^	**0.005**	**0.378**	**0.193**	**0.740**	0914	0.969	0.551	1.704
Pastoralists^b^	0.466	0.788	0.415	1.496	0.800	0.929	0.526	1.640
Urban admin^c RC^								
Place of residence								
Urban	0.105	0.498	0.215	1.158	0.840	1.057	0.615	1.816
Rural ^RC^								
Educational status								
Not Educated	0.740	1.080	0.685	1.705	0.139	0.778	0.558	1.085
Primary and above ^RC^								
Religion								
Orthodox Christian	0.798	1.073	0.624	1.847	**0.010**	**1.698**	**1.137**	**2.536**
Muslim	0.889	0.964	0.572	1.623	0.423	1.181	0.786	1.777
Others^d RC^								
Number of family members								
1–4	0.845	0.939	0.479	1.839	0.130	1.498	0.888	2.527
5–7	0.590	0.875	0.538	1.422	0.121	1.380	0.918	2.074
> = 8 ^RC^								
Sex of household head								
Male	0.592	0.868	0.518	1.456	0.754	0.937	0.622	1.411
Female^RC^								
Wealth Index								
Poor	0.154	0.696	0.423	1.146	**0.007**	**0.608**	**0.424**	**0.873**
Middle	0.466	1.216	0.718	2.061	0.257	0.791	0.527	1.187
Rich ^RC^								
Ever used anything to delay pregnancy								
No	**0.005**	**0.528**	**0.337**	**0.826**	**0.030**	**0.694**	**0.499**	**0.966**
Used outside calendar	0.410	1.396	0.631	3.091	0.466	1.264	0.673	2.376
Used in calendar ^RC^								
Wanted Pregnancy when become pregnancy								
Then	0.465	1.388	0.576	3.345	**0.031**	**0.579**	**0.352**	**0.953**
Later	0.051	2.544	0.998	6.486	0.894	1.038	0.597	1.805
No more ^RC^								
Number of ANC Visits								
Have no ANC visit	**0.000**	**0.245**	**0.145**	**0.413**	**0.000**	**0.264**	**0.184**	**0.380**
1–3 visits	0.826	0.955	0.634	1.439	**0.001**	**0.568**	**0.412**	**0.784**
> =4 visits ^RC^								
Marital status								
Married/lived with partner.	0.745	1.156	0.483	2.770	0.876	0.955	0.535	1.705
Others^e RC^								
Participant’s occ.								
No work	0.225	0.745	0.462	1.200	0.065	0.711	0.495	1.022
Agricultural work	0.446	0.812	0.475	1.387	0.999	1.000	0.672	1.489
Others ^fRC^								
Ever heard of fistula								
No	0.064	0.697	0.476	1.021	0.264	0.844	0.627	1.136
Yes ^RC^								
Number of living children								
4 and bellow	0.3688	0.692	0.310	1.544	0.200	1.423	0.830	2.440
5 and above ^RC^								
Frequency of listing radio								
Not at all	0.196	0.691	0.394	1.210	**0.041**	**0.652**	**0.432**	**0.984**
Less than once a week	0.606	0.933	0.416	1.668	0.235	0.731	0.435	1.227
At least once a week ^RC^								
Owns mobile telephone								
No	0.298	1.376	0.755	2.511	0.311	0.806	0.530	1.224
Yes ^RC^								
Has bank account								
No	**0.033**	**0.479**	**0.243**	**0.943**	0.642	0.886	0.532	1.476
Yes ^RC^								
Ever born children								
1–2 children	0.789	0.876	0.333	2.305	0.152	0.622	0.325	1.192
3–4 children	0.827	1.094	.489	2.449	0.299	0.757	0.448	1.280
> = 5 children ^RC^								
Size of the newborn at birth								
Larger than average	0.785	0.934	0.573	1.524	0.374	0.845	0.583	1.225
Average	0.676	1.097	0.711	1.693	0.908	0.981	0.708	1.358
Smaller than average								

^a^ Tigray, Amhara, Oromo, and South nation nationality and peoples. ^b^Afar, Gambella, Benishangule, and Somalia, ^c^Addis-Ababa, Harrar and Drie-Dawa. ^d^Catholic, Protestant and Others. ^e^ Single, divorced, widowed, separated. ^f^Daily laborer, industrial work and others.

*AOR* Adjusted Odds Ratio. *RC* Reference Category

Boldfaces significant variables at *p* < 0.05

Similar to the findings on factors associated with receiving PNC at home, there were a number of factors associated with receiving PNC at a facility among women who delivered at home. Religion, wealth index, ever using anything to delay pregnancy, wantedness of the pregnancy, ANC visits and listening to radio were associated with facility-based PNC utilization. Women who follow the Orthodox religion were 69% more likely (AOR = 1.698, 95%CI 1.137–2.539) to utilize PNC at a health facility than not utilizing at all when compared to women whose religion was “others”(not orthodox or Muslim). Mothers in the low wealth index were 40% less likely (*AOR* = 0.608, 95%CI 0.424–0.873) to utilize PNC at a health facility than not utilizing at all when compared to women in the upper wealth index. Women who wanted their pregnancy were 42% less likely (*AOR* = 0.579, 95%CI 0.352–0.953) to utilize PNC at a health facility than not utilizing at all when compared with women who had not want to became pregnant. Lastly, listening to radio was associated with PNC utilization in a health facility; women who did not listen to radio at all were 34.8% less likely (*AOR* = 0.652, 95%CI 0.432–0.984) to utilize PNC at a health facility than not utilizing at all when compared with women who listened to radio at least once a week when other variables were held constant (Table 4).

## Discussion

This study used data from the 2016 DHS assessment in Ethiopia-the fourth survey to capture detailed information on maternal and newborn health in general and PNC services in particular at national level. In this paper we have explored the coverage of home-based and facility-based PNC utilization among mothers who delivered at home, and factors associated with place of PNC service utilization in Ethiopia. Below we will discuss the implications of the study findings for program, policy and future research in Ethiopia.

This study shows that only 8.2% of mothers who delivered at home utilized PNC services (2.9% of mothers received at home and 5.3% a health facility). Analysis of the factors associated with place of PNC utilization in the study show that region type, religion, wealth index, having a bank account, ever using anything to delay pregnancy, wantedness of pregnancy, antenatal care visits, and radio listening appear to be important factors for PNC utilization either at home or facility in Ethiopia.

In this study, the magnitude of PNC utilization either at home or at a health facility was similar with findings from some studies [1, 24–26] though lower than the findings from other studies [11, 13, 27–30]. This difference could be due to differences in the schedule of visits considered in the different studies, which could affect the comparability and results between the different studies [1]. Additionally, it could reflect differences in the socio-demographic and socio-economic characteristics of the study populations. Furthermore, these studies may have included PNC given at a health facility to women who delivered in health facilities, which would be expected to be higher compared to a study like ours’, which only considered mothers who delivered at home.

In our study, mothers from urban regions were more likely to utilize PNC at home compared to participants from agrarian regions while there was no statistically significant variation in PNC utilization in a facility by region type. Being from urban regions (Addis Ababa, Dire-Dawa, and Harrar) could expose women to improved awareness and good access to trained professionals [24, 31] even to mobile home-based care. Utilization of services that precede PNC, including ever using anything to delay pregnancy and receiving ANC were two other factors which were significantly associated with PNC service utilization, both at home and at a health facility. This is not surprising, as many other studies [28–30, 32–37] found similar findings. It is plausible that if mothers had previous contact with health professionals (especially for ANC), they would be more likely to seek PNC at a health facility or to have contact with a community health worker after giving birth to receive PNC at home [1, 32, 38].

Surprisingly, although the vast majority of women wanted the pregnancy at the time they became pregnant (80%, *n* = 3605), women who did not wish to become pregnant then were more likely to utilize PNC at health facilities compared to those who wanted the pregnancy when they become pregnant. This may be related a higher incidence of complications developing after birth among those with unwanted pregnancies. Additionally, findings from other studies have revealed that mothers may not seek care at health facilities if they did not develop complications [1, 18, 39, 40], and those who did not want the pregnancy may develop more complications than those who wanted it.

Media exposure was also associated with PNC service utilization, women who listened to radio at least once a weak were more likely to utilize PNC at a health facility compared to those who did not listen to radio at all. Media exposure could improve the awareness of women of the value of seeking care [41], including PNC, even among mothers who gave birth at home.

Furthermore, mothers with a bank account were more likely to utilize PNC at home than those without bank account, and mothers from the highest wealth index were more likely to receive PNC at a health facility compared to women in the lowest wealth index. These findings are similar with other findings [31, 42–44]. A possible pathway of the influence of these factors could be that mothers with the lowest income could face financial hardship, including in obtaining transport to a health facility, while those with the highest income may struggle less with transport coast. In this study, mothers who follow the Orthodox Christian religion were more likely to utilize PNC at a health facility when compared with women of other religions, excluding Muslim women. Though we could not find any reference to explain this finding, a possible reason could be that there may be differences in health facility access among the Orthodox Christian followers compared to other religious groups (excluding Muslim women). Furthermore, there could be differences in the involvement of religious leaders in advising followers to utilize modern health services. However, this needs further research to explore the differences in PNC utilization among followers of different religions.

### Limitations and strengths of the study

This study made use of cross-sectional data from the 2016 Ethiopian Demographic and Health Survey. The data relies on women’s self-reported care utilization, and may be influenced by recall bias, given that the study events took place within the 5 years preceding the survey.

However, the study has a number of strengths. The data is national survey data, and the sample size is powered to be generalizable at national and regional level. This study was unique in that conducted advanced analysis of PNC coverage and location, and factors associated with place of PNC service utilization among mothers who delivered at home. These findings will provide useful information for practitioners and policy makers for increasing utilization of PNC among the substantial population of mothers who give birth at home. It also highlights research areas that need further studies in the future to better understand the barriers and facilitators of place of PNC.

## Conclusion

The utilization of postnatal care service among mothers who delivered at home is low in Ethiopia. Living in urban regions, having a bank account, and ever using anything to delay pregnancy were associated with receiving PNC at home. Furthermore being in the upper wealth index, having unwanted pregnancy, receiving 4+ ANC visits and listening to radio weekly were associated with PNC utilization in health facilities among women who delivered at home. Therefore, targeted measures to improve socio-economic status, strengthen the continuum of care and improve health literacy communication are critically important to mitigate the gaps on postnatal care service utilization among women who deliver at home.

## Data Availability

The dataset generated and/or analyzed for the current study is available from MEASURE DHS project but restrictions apply to the availability of the data, which were used under license for the current study, and so are not publicly available. However data is available from the MEASURE DHS project upon reasonable online request.

## Abbreviations

ANC: Antenatal Care
AOR: Adjusted Odds Ratio
EDHS: Ethiopian Demographic Health Survey
PNC: Postnatal Care
WHO: World Health Organization
